# Diagnosis of TIA (DOT) score – design and validation of a new clinical diagnostic tool for transient ischaemic attack

**DOI:** 10.1186/s12883-016-0535-1

**Published:** 2016-02-09

**Authors:** Dipankar Dutta

**Affiliations:** Stroke Service, Gloucestershire Royal Hospital, Great Western Road, Gloucester, GL1 3NN UK

**Keywords:** Transient ischaemic attack, Diagnosis, Diagnostic score, Logistic regression

## Abstract

**Background:**

The diagnosis of Transient Ischaemic Attack (TIA) can be difficult and 50–60 % of patients seen in TIA clinics turn out to be mimics. Many of these mimics have high ABCD2 scores and fill urgent TIA clinic slots inappropriately. A TIA diagnostic tool may help non-specialists make the diagnosis with greater accuracy and improve TIA clinic triage. The only available diagnostic score (Dawson et al) is limited in scope and not widely used. The Diagnosis of TIA (DOT) Score is a new and internally validated web and mobile app based diagnostic tool which encompasses both brain and retinal TIA.

**Methods:**

The score was derived retrospectively from a single centre TIA clinic database using stepwise logistic regression by backwards elimination to find the best model. An optimum cutpoint was obtained for the score. The derivation and validation cohorts were separate samples drawn from the years 2010/12 and 2013 respectively. Receiver Operating Characteristic (ROC) curves and area under the curve (AUC) were calculated and the diagnostic accuracy of DOT was compared to the Dawson score. A web and smartphone calculator were designed subsequently.

**Results:**

The derivation cohort had 879 patients and the validation cohort 525. The final model had seventeen predictors and had an AUC of 0.91 (95 % CI: 0.89–0.93). When tested on the validation cohort, the AUC for DOTS was 0.89 (0.86–0.92) while that of the Dawson score was 0.77 (0.73–0.81). The sensitivity and specificity of the DOT score were 89 % (CI: 84 %–93 %) and 76 % (70 %–81 %) respectively while those of the Dawson score were 83 % (78 %–88 %) and 51 % (45 %–57 %). Other diagnostic accuracy measures (DOT vs. Dawson) include positive predictive values (75 % vs. 58 %), negative predictive values (89 % vs. 79 %), positive likelihood ratios (3.67 vs. 1.70) and negative likelihood ratios (0.15 vs. 0.32).

**Conclusion:**

The DOT score shows promise as a diagnostic tool for TIA and requires independent external validation before it can be widely used. It could potentially improve the triage of patients assessed for suspected TIA.

**Electronic supplementary material:**

The online version of this article (doi:10.1186/s12883-016-0535-1) contains supplementary material, which is available to authorized users.

## Background

The diagnosis of transient ischaemic attack (TIA) can be difficult and studies show limited inter-observer agreement for clinical diagnosis [1]. About 50 to 60 % of TIA referrals by non- specialists turn out to be non- cerebrovascular mimics [2–4]. Patients with TIA have a high risk of early stroke and subsequent adverse events [5, 6]. Following secondary prevention studies [7, 8] and the introduction of the ABCD2 score [9], rapid assessment TIA clinics have been set up to investigate and manage TIA. Inappropriate referrals to TIA clinics, however, can lead to delays for patients with TIA and the misdiagnosis of non -cerebrovascular conditions as TIA leads to unnecessary anxiety and inappropriate initial management.

Stroke diagnostic tools such as FAST and ROSIER have been developed for use by pre hospital assessors and emergency room clinicians [10, 11]. The ABCD2 score, too, has been used as a crude diagnostic aid for TIA [12]. More recently, the ability of the ABCD2 score to reliably discriminate between those at high or low risk after a TIA has been called into question and a third of mimics found to have ABCD2 scores ≥ 4 [13]. A TIA diagnostic tool could be used to improve TIA clinic triage by removing some mimics from urgent TIA pathways. There is only one TIA diagnostic tool, the score of Dawson and colleagues [14] which was not designed for retinal and some posterior circulation events and is not widely used. It has shown limited accuracy when used in a primary care setting [15]. The Diagnosis of TIA Score (DOTS) is a new tool to help non-specialists make the diagnosis of TIA with greater accuracy. It includes retinal and posterior circulation events and is meant for use as a mobile app and web based calculator.

## Methods

### Development cohort

The development cohort for the score was a subset of TIA clinic patients studied retrospectively from a TIA database [2]. Briefly, all patients referred to the Monday to Friday TIA clinics of Gloucestershire Royal Hospital (GRH), Gloucester, UK between April 2010 and May 2012 were eligible for inclusion in the development cohort. The catchment area for GRH has a population of 560,000. Referrals are accepted from Emergency Departments, General Practitioners, paramedics and other departments such as ophthalmology.

Data collected included demographic information, past medical history, a detailed history, examination findings, ABCD2 scores, results of investigations (blood tests, ECG, same day carotid duplex ultrasounds, same day CT brain scans) and final diagnosis [2]. MRI scans were not done on the same day but later as required. The diagnosis was made by consultant stroke physicians with at least 7 to 10 years of stroke experience. Patients were classified as TIA, minor stroke or mimic. TIA was defined as an acute loss of focal cerebral or ocular function lasting < 24 h and presumed to be caused by embolic or thrombotic vascular disease while a stroke was diagnosed if symptoms lasted > 24 h [16, 17]. A diagnosis of stroke was also made if symptoms lasted < 24 h but there was a new infarct visible on CT [18]. The minor strokes in this cohort were patients who had minimal symptoms or signs and did not require hospital admission. TIAs and strokes included retinal as well as cerebral events. The traditional NINDS diagnostic criteria [19] for TIA were used with a few exceptions at the discretion of the diagnosing physician. In the absence of a gold standard for diagnosis and lack of same day diffusion weighted MRI, to make the clinic diagnosis more robust, follow up data for a median of 34.9 months (IQR 27.7 – 41.6) were accessed to look at subsequent vascular events and death [2]. In a small number of cases, the final diagnosis was altered based on new information from follow up.

### Ethics, consent and permissions

The study had the necessary institutional permission from the Gloucestershire Hospitals NHS Foundation Trust (GHNHSFT). It was reviewed by the funding Research and Innovation Forum of the GHNHSFT and Gloucestershire Research Support Service (GRSS) with regards to the appropriate IRB/Ethical reviews necessary. The study was assessed as requiring no Research Ethics Committee/IRB review or formal patient consent. The basis for this decision, as per the regulations of the UK Health Departments Governance Arrangements for Research Ethics Committees, was that the study was limited to secondary use of information previously collected in the course of normal clinical care and that the no patient identifiers were recorded in the dataset for analysis. No patient contact or additional procedures were necessary for this study.

### Statistical analysis

#### Selection of variables for analysis

Information derived from the history were coded into discrete binary variables such as “unilateral weakness”, “dysphasia”, “dysarthria”, “headache”, “amnesia” etc. These variables were selected from clinical features expected to predict stroke or TIA based on previous experience as well as those likely to favour mimics such as migraine and seizures [10, 11, 14, 19, 20]. Age and risk factors were other potential predictors. Preliminary univariate analysis was used to identify predictive variables although non- significant variables were not excluded automatically from fitted models.

#### Logistic regression and model selection

The dependent variable was “definite cerebrovascular disease” which included TIAs and minor strokes of the brain or eye. Stepwise multiple logistic regression using the backwards elimination method was performed to select the best model using the Akaike information criterion [21].

#### The actual score

The diagnostic score was derived from the coefficients of the final model and the intercept [21]. Calibration of the models was tested by calibration plots and the Hosmer-Lemeshaw statistic and discrimination by Receiver Operating Characteristic (ROC) curves and area under the curve (AUC or c statistic) [22]. Optimal cutpoints for the score were derived from the ROC curve using two methods; criteria based on sensitivity and specificity alone using the Youden Index and the cost of misclassification method (cost benefit analysis of diagnosis) where an assumption of a 2:1 cost ratio was made (i.e. the cost of misclassifying a TIA as a mimic is twice that of misclassifying a mimic as a TIA) [23]. Specificity, sensitivities and other measures of diagnostic accuracy were calculated for each cut point [24]. A web based calculator and smartphone app were subsequently designed to calculate the logit, probability of the outcome and to present the result as “probable TIA”, “possible TIA” or “TIA unlikely.

Although no formal sample size calculation was undertaken, the rule of thumb to satisfy the requirements for developing a score by logistic regression modelling was met by using the available sample; there were more than 10–20 outcome events per potential predictor variable studied [25, 26].

#### Internal validation

A separate validation cohort of patients was taken from patients referred to the GRH TIA clinic from January to August 2013. Baseline characteristics of the derivation and validation cohorts were compared using the *t*-test and chi-squared test. The DOTS and Dawson score [14] were calculated retrospectively by one observer blinded to the clinic diagnosis. The clinic diagnosis, made by a stroke consultant, was accepted as the gold standard. Predicted and observed diagnoses were plotted to test calibration and discrimination was tested by the c statistic with 95 % CI. The c statistic for ABCD2 scores (as recorded by the referring clinicians) and Dawson scores were also calculated and ROC curves compared. Data were analysed using “R” [27].

## Results

The derivation dataset was a subset of 1067 patients [2]. Of the 1067 records, 188 records were rejected as the initial history had been recorded by trainee doctors or specialist nurses and not experienced stroke consultants. Data for 879 patients were satisfactory in every respect and used as the training dataset. In 12 out of 879 patients, the final diagnosis was altered based on new information from follow up.

The validation cohort comprised 525 separate patients referred between January and August 2013. Table 1 shows the baseline characteristics of the development and validation datasets. Of patients with stroke or TIA in the development cohort, 65 % had anterior circulation events, 16 % posterior circulation and 19 % retinal events. The validation cohort had 70 % anterior circulation, 13 % posterior and 17 % retinal events. The two cohorts were different in many respects including the proportion of minor strokes, presence of previous vascular disease, hypertension, diabetes and atrial fibrillation (AF).

**Table 1:** Characteristics of the developmental and validation datasets

	Development cohort (*n* = 879)	Validation cohort (*n* = 525)	*P* values
Age in years (mean, SD(standard deviation)	69.3 (13.8)	70.8 (14.0)	0.051
Sex (proportion of females)	50.4 %	54.1 %	0.198
Referred by:	Not available		N/A
GP		66.1 %	
ED		18.1 %	
Other		15.8 %	
Proportion of TIA	272 (30.9 %)	160 (30.5 %)	0.901
Proportion of stroke	174 (18.7 %)	76 (14.5 %)	0.014
Proportion of mimics	443 (50.4 %)	289 (55.1 %)	0.103
Previous cerebrovascular disease	128 (14.6 %)	32 (6.1 %)	< 0.001
Hypertension	365 (41.5 %)	106 (20.2 %)	< 0.001
Diabetes	117 (13.3 %)	27 (5.2 %)	< 0.001
AF	101 (11.2 %)	25 (4.8 %)	< 0.001
Smoker	165 (18.8 %)	24 (4.6 %)	< 0.001

*GP*, general practitioner, *ED*, emergency department

The initial logistic regression model included the following predictors; age, sex, AF, hypertension, previous cerebrovascular disease, diabetes, other vascular disease (including ischaemic heart disease and peripheral vascular disease), dysphasia, dysarthria, facial weakness, unilateral weakness (arm, leg or both), sensory loss, monocular visual loss, diplopia, bilateral visual loss, hemianopia, visual aura (scintillations, fortification spectra or spreading scotoma), dizziness, vertigo, ataxia (either limb, gait or both), headache, confusion, tingling/numbness (face or limbs), amnesia, seizure (defined as any rhythmical involuntary movement) and loss of consciousness or near loss of consciousness. After the stepwise selection process, during which 9 different models were assessed, the following variables were left in the final model: age, history of hypertension, history of AF/new onset AF and fourteen clinical features as shown in Table 2, which lists the coefficients, standard errors odds ratios with 95 % confidence intervals of each predictor in the model. The intercept of the final model was -3.365. All the variables were significant at a level of *p* <0.05 (many significant at *p* <0.001) except for hypertension. The only continuous predictor was age and an assumption of linearity was made for this predictor. The optimal cut point for the score was 0.297 using the Youden Index and – 0.547 using the cost of misclassification method. The calculated sensitivity using a cutpoint of 0.297 was 84 % and specificity 85 % while the cutpoint – 0.547 gave a sensitivity of 93 % and specificity of 74 %. This cutpoint (-0.547) was the one used in the final score and calculators to differentiate between non-TIA and possible or probable TIA. Patients with a DOT score ≥ 0.297 (probability of TIA > 57.4 %) were classified as “Probable TIA” with those between -0.547 and 0.297 classified as “Possible TIA” and those with a DOT score of < - 0.547 (probability of TIA < 36.7 %) were classed as “TIA unlikely” (Additional file 1).

**Table 2:** Predictors in the final model with coefficients, standard errors (SE), Odds Ratios and 95 % Confidence Intervals. The intercept was -3.365

Predictor	Regression coefficients (SE)	Odds Ratios	95 % CI
Age	0.02 (0.01)	1.0	1.0–1.03
History of hypertension	0.32 (0.20)	1.4	0.9–2.0
History of AF or new AF	0.62 (0.32)	1.7	1.0–3.5
Dysphasia	3.13 (0.30)	22.9	12.8–42.5
Unilateral facial weakness (UMN)	1.69 (0.35)	5.4	2.7–11.0
Unilateral weakness (arm, leg or both)	3.15 (0.28)	23.3	13.6–41.2
Monocular visual loss	3.58 (0.35)	35.8	18.3–73.2
Diplopia	2.14 (0.56)	8.5	2.9–26.4
Bilateral visual loss	1.83 (0.52)	6.2	2.2–17.2
Hemianopia	3.25 (0.56)	25.8	9.0–81.1
Visual aura (fortification spectra, scintillations or spreading scotoma)	−1.84 (0.35)	0.2	0.1–0.3
Unilateral sensory loss	2.11 (0.95)	8.2	1.4–67.4
Ataxia (limb or gait)	2.06 (0.37)	7.9	3.9–16.6
Headache	−0.66 (0.25)	0.5	0.3–0.8
Amnesia	−1.70 (0.62)	0.2	0.04–0.6
Loss of consciousness or pre-syncope	−0.78 (0.39)	0.5	0.2–1.0
Tingling/numbness/pins and needles	−0.80 (0.26)	0.5	0.3–0.7

**Fig. 1:**
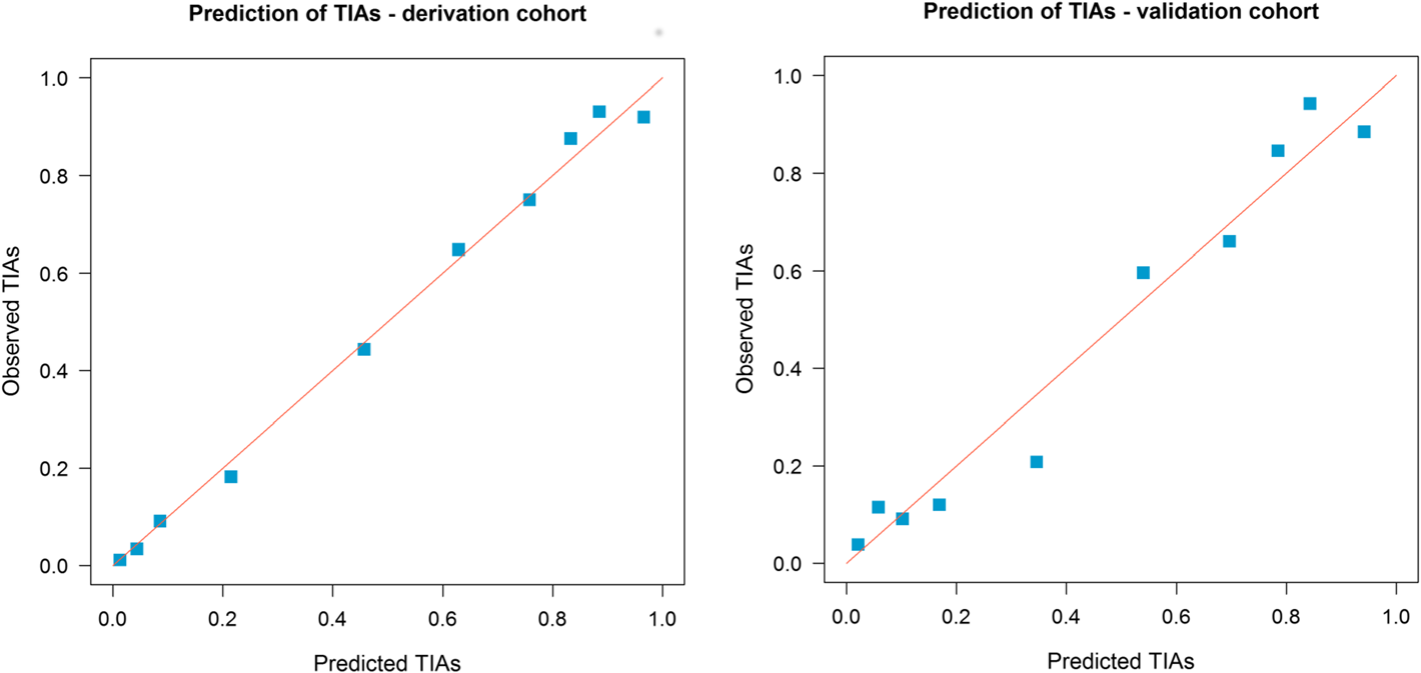
Calibration plots for the final DOT model on the derivation and validation cohorts

Figure 1 shows the calibration plots (predicted vs observed results) of the final model on the derivation and validation cohorts. The *P* value was 0.273 for the Hosmer-Lemeshow test of goodness of fit in the derivation sample. As would be expected, the plots show better calibration for the derivation than validation datasets.

**Fig. 2:**
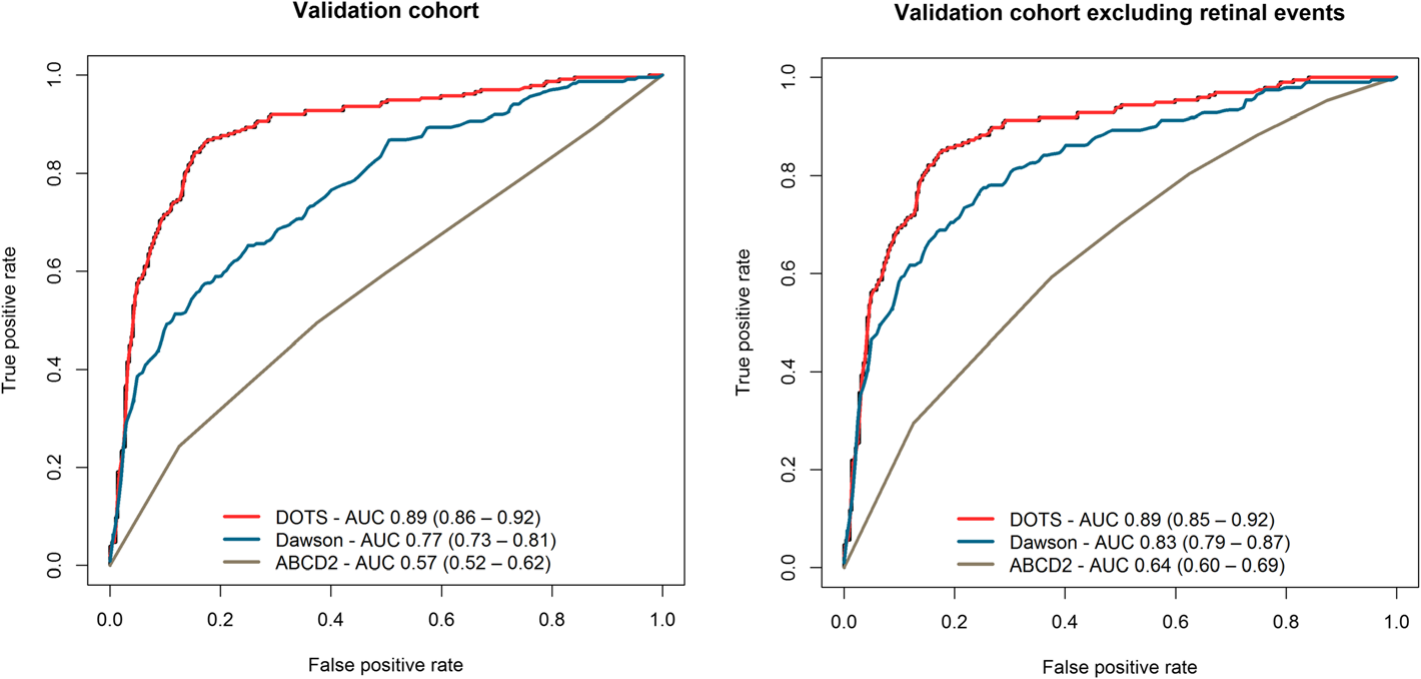
ROC curves for the DOT, Dawson and ABCD2 scores on the full validation cohort (*n* = 525) and on the validation cohort excluding retinal events (*n* = 485)

The c statistic (AUC) was 0.91 (95 % CI: 0.89 - 0.93) for the final model on the derivation cohort. Figure 2 compares the ROC curves drawn to assess discrimination of the DOTS, Dawson and the ABCD2 scores when applied to the full validation cohort and the validation cohort excluding retinal events (*n* = 485). The c statistic with confidence intervals are shown. Both the DOT and Dawson scores showed good discrimination.

Table 3 shows the actual numbers of positive and negative diagnoses and several diagnostic accuracy measures using the DOT score (with two alternative cutpoints) and the Dawson score tested on the validation dataset. As the Dawson score was not originally designed for retinal events, all test parameters for the DOT and Dawson scores were repeated using the validation dataset excluding retinal vascular events (*n* = 485). These results are also shown in Table 3.

**Table 3:** Diagnostic accuracy of DOT (cutpoint 0.297), DOT (cutpoint – 0.547) and Dawson scores on full validation cohort and cohort excluding retinal events. Confidence intervals (95 %) are shown where available

Full validation cohort (*n* = 525)			
	DOT ^0.297^	DOT ^-0.547^	Dawson
True positive	192	210	197
False positive	41	70	142
True negative	248	219	147
False negative	44	26	39
Sensitivity	81 % (76 %–86 %)	89 % (84 %–93 %)	83 % (78 %–88 %)
Specificity	86 % (81 %–90 %)	76 % (70 %–81 %)	51 % (45 %–57 %)
Positive predictive value	82 % (77 %–87 %)	75 % (70 %–80 %)	58 % (53 %–63 %)
Negative predictive value	85 % (80 %–89 %)	89 % (85 %–93 %)	79 % (72 %–85 %)
Positive likelihood ratio	5.73 (4.29–7.66)	3.67 (2.98–4.53)	1.70 (1.49–1.94)
Negative likelihood ratio	0.22 (0.17–0.28)	0.15 (0.10–0.21)	0.32 (0.24–0.44)
Validation cohort excluding retinal events (*n* = 485)			
	DOT ^0.297^	DOT ^-0.547^	Dawson
True positive	156	172	175
False positive	41	70	142
True negative	248	219	147
False negative	40	24	21
Sensitivity	80 % (73 %–85 %)	88 % (82 %–92 %)	89 % (84 %–93 %)
Specificity	86 % (81 %–90 %)	76 % (70 %–81 %)	51 % (45 %–57 %)
Positive predictive value	79 % (73 %–85 %)	71 % (65 %–77 %)	55 % (50 %–61 %)
Negative predictive value	86 % (82 %–90 %)	90 % (86 %–94 %)	88 % (82 %–92 %)
Positive likelihood ratio	5.61 (4.19–7.51)	3.62 (2.94–4.47)	1.82 (1.60–2.06)
Negative likelihood ratio	0.24 (0.18–0.31)	0.16 (0.11–0.24)	0.21 (0.14–0.32)

In the 24 patients in whom the DOT score wrongly missed TIAs or strokes, 15 were anterior circulation events, eight posterior circulation and one was a retinal TIA. However, only 7/24 (29 %) had some imaging (CT, MR or carotid) abnormalities in keeping with cerebrovascular disease. Many of the histories in this group of patient were atypical with non -focal as well as focal features and the diagnoses were recorded as probable or possible TIA /stroke suggesting some diagnostic uncertainty.

## Discussion

The DOT score is a new TIA diagnostic tool which performed well in comparison to the Dawson score [14] when applied to the validation dataset. The sensitivities were very similar in the two scores but other measures such as specificity, positive and negative predictive values and AUC were superior for DOTS. Unlike the Dawson score, this score attempts to encompass the entire spectrum of TIA/stroke that would be expected in a TIA clinic including brain (anterior and posterior circulation) and retinal events. In contrast to a previous study, the ABCD2 scores, which were those recorded by the referring clinician, showed very poor discrimination for the diagnosis of TIA [12]. This suggests, in keeping with a recent meta-analysis [13], that clinic triage based on non-specialist use of the ABCD2 score could be improved by the use of a diagnostic score to enable quicker assessment of patients with a higher a priori probability of TIA or minor stroke.

Seizure and dysarthria are two seemingly surprising omissions from the final model. There were only 18 patients with overt rhythmical movements in the derivation cohort (of which at least one had limb shaking TIA) and other potentially postictal features like amnesia and loss of consciousness were significant. In contrast to some other scores [10, 11, 14], the DOT score attempts to distinguish between dysphasia and dysarthria to improve its specificity. It is well known that dysarthria has a broader differential diagnosis and, particularly in isolation, does not necessarily suggest a TIA. Other common cerebrovascular symptoms which are often associated with dysarthria were significant in the final model suggesting that the omission of dysarthria may not affect the sensitivity of the score. Many patients with transient monocular visual loss in the derivation cohort had been referred by the ophthalmology department and so alternative diagnoses may have been screened out leading to a higher preponderance of ocular TIA in this group of patients. The recommendation that all patients with visual loss should have other ocular pathology excluded before attributing the visual loss to a TIA, therefore extends to the presumed diagnosis of retinal TIA based on this score. It is also necessary to emphasise that patients with ongoing neurological signs or symptoms or any appropriate lesion on imaging should be considered as having a stroke and managed accordingly.

It has been suggested that it is unrealistic for a clinical scoring system to cover all types of TIA given the heterogeneity of their symptoms [14]. However, this score was derived from a TIA cohort with a typical case-mix of anterior, posterior and retinal events as well as a high proportion of mimics. This explains why the score is not “parsimonious” and includes 17 items; more predictors are needed to sort TIA from mimics given their varied symptoms. A model as complex as this can be considered impractical but becomes highly usable when presented as a calculator or mobile app. Once the history is taken, using the web calculator or app takes about 30 s. This approach also enables guidance notes to be incorporated in the tool to help non- specialist assessors select the appropriate predictors with greater accuracy. It is well known that predictive tools derived in one health care setting may not translate well to others. It is hoped, however, that the guidance provided will preserve the score’s predictive performance when it is used by primary care or emergency department physicians.

The sample size used to derive the DOT score may not be considered large but it met the essential requirements of developing a score by logistic regression modelling [25, 26]. Although same day DWI-MR was not available, diagnosis was made by experienced stroke physicians mostly using standard NINDS diagnostic criteria [19]. Results of follow up were taken into account to refine the diagnoses made. The dataset for the derivation cohort was complete in every respect. Validation, although internal and retrospective used a separate set of patients from a different time point which differed in many respects from the derivation cohort. The DOT and Dawson scoring was done by one observer blinded to the clinic diagnosis. The difference in the two cohorts was probably due to chance alone and strengthens the external validity of the DOT score. The case-mix suggests that the score should be generalizable to all TIA services which accept unselected patients referred by primary care physicians, emergency or other departments.

## Conclusion

In conclusion, the DOT score shows promise as an useful tool for the diagnosis of TIA which will require external validation before it can be widely used. Impact studies would also be necessary to show that the score improves TIA clinic triage.

## Additional file

Additional file 1:
**Online supplement DOTS Calculator.** (html 20.5 kb)
