# Supplementary material for: Diagnosis of TIA (DOT) score – design and validation of a new clinical diagnostic tool for transient ischaemic attack
Source: BMC Neurol. 2016 Feb 9;16:20. doi: 10.1186/s12883-016-0535-1 (PMC4746899; doi:10.1186/s12883-016-0535-1)
Supplement: Additional file 1: — Online supplement DOTS Calculator. (html 20.5 kb) [file 12883_2016_535_MOESM1_ESM.html]

DOTS Calculator


# Diagnosis of TIA Score Calculator

The DOT score is a diagnostic tool designed to help non- specialists diagnose a transient ischaemic attack (TIA) or minor stroke with greater accuracy.This score is currently for research purposes only and has not yet been externally validated.

## Instructions for use

Enter age of patient in years and check each box that applies to your patient. Please take a thorough history and enter each item as accurately as possible. Then click the 'Calculate DOTS' button. Click the 'Reset' button to clear the form.

# Data Entry

| Item | Input | Explanatory notes |
| --- | --- | --- |
| Age |  | Enter age in years. |
| History of hypertension |  | Select if patient has a history of hypertension even if recently diagnosed. |
| Atrial fibrillation (AF or PAF) |  | Select if patient has known AF,PAF or atrial flutter or has just been found by you to be in AF. |
| Dysphasia (disorder of language) |  | Select ONLY if patient had word finding difficulties, jumbled speech or was unable to speak. Slurring of speech (dysarthria) does NOT count as dysphasia. |
| Unilateral facial weakness |  | Select if patient had unilateral upper motor neurone (forehead sparing) facial weakness. If patient has isolated facial weakness at present and it is a lower motor neurone weakness, consider Bells Palsy. |
| Unilateral weakness of arm, leg or both |  | This must be GENUINE weakness. Tingling, numbness, heaviness, deadness or pain does NOT count unless there was true weakness. Ask if it was difficult to move the limb or grip. |
| Unilateral sensory loss |  | This must be genuine LOSS of sensation. Tingling, numbness or deadness does NOT count unless patient is sure there was loss of pain, temperature or touch sensation. |
| Visual loss in one eye |  | Either partial or complete monocular blindnes. Check if patient is sure it was one eye - did they close each eye in turn? Transient loss can be due to a TIA affecting the eye. Persistent visual loss can have a broader differential diagnosis and in all cases, an ophthalmology review is required. |
| Visual loss in both eyes |  | Applies to complete blindness affecting both eyes. |
| Diplopia |  | Double vision.Does NOT apply to non specific blurring of vision. |
| Homonymous hemianopia |  | Applies to visual loss in either the right or left visual field.Please do not mistake this for monocular blindness or vice versa. |
| Visual aura |  | Applies to scintillations (flashing lights), fortification spectra (zig-zag lines) or spreading scotoma as in a migraine type visual aura. |
| Ataxia |  | Applies to inco-ordination of the limbs or gait. |
| Headache |  | Applies to any headache before,with or after the episode. |
| Amnesia |  | Does the patient remember the episode? Do not select if patient has dementia and is unlikely to remember what happened. |
| Loss of consciousness or near LOC |  | This applies to loss of consciousness due to any reason or near LOC. |
| Tingling and numbness |  | This applies to tingling, numbness or pins and needles to any part of the body including face. |
| Evaluate  |  | |

Designed by Dr Dipankar Dutta, Gloucestershire Royal Hospital, Gloucester, UK. November 2015.

Reference:Dutta D. Diagnosis of TIA (DOT) score – design and validation of a new clinical diagnostic tool for transient ischaemic attack. BMC Neurology. DOI: 10.1186/s12883-016-0535-1

DISCLAIMER: This calculator is for the use of qualified medical personnel in the context of research. It has not yet been externally validated. It is not a substitute for clinical assessment by a stroke neurologist or physician.
